# High-Performance Resistive Pressure Sensor Based on Elastic Composite Hydrogel of Silver Nanowires and Poly(ethylene glycol)

**DOI:** 10.3390/mi9090438

**Published:** 2018-08-30

**Authors:** Youngsang Ko, Dabum Kim, Goomin Kwon, Jungmok You

**Affiliations:** 1 Department of Plant & Environmental New Resources, Kyung Hee University, 1732 Deogyeong-daero, Giheung-gu, Yongin-si, Gyeonggi-do 446-701, Korea; ysko1119@naver.com (Y.K.); kdb511@naver.com (D.K.); 2 Graduate School of Biotechnology, Graduate School, Kyung Hee University, 1732 Deogyeong-daero, Giheung-gu, Yongin-si, Gyeonggi-do 446-701, Korea; gracegm@hanmail.net

**Keywords:** silver nanowire (AgNW), hydrogel, PEG photolithography, nanocomposite, electronics, pressure sensor

## Abstract

Improved pressure sensing is of great interest to enable the next-generation of bioelectronics systems. This paper describes the development of a transparent, flexible, highly sensitive pressure sensor, having a composite sandwich structure of elastic silver nanowires (AgNWs) and poly(ethylene glycol) (PEG). A simple PEG photolithography was employed to construct elastic AgNW-PEG composite patterns on flexible polyethylene terephthalate (PET) film. A porous PEG hydrogel structure enabled the use of conductive AgNW patterns while maintaining the elasticity of the composite material, features that are both essential for high-performance pressure sensing. The transparency and electrical properties of AgNW-PEG composite could be precisely controlled by varying the AgNW concentration. An elastic AgNW-PEG composite hydrogel with 0.6 wt % AgNW concentration exhibited high transmittance including T_550nm_ of around 86%, low sheet resistance of 22.69 Ω·sq^−1^, and excellent bending durability (only 5.8% resistance increase under bending to 10 mm radius). A flexible resistive pressure sensor based on our highly transparent AgNW-PEG composite showed stable and reproducible response, high sensitivity (69.7 kPa^−1^), low sensing threshold (~2 kPa), and fast response time (20–40 ms), demonstrating the effectiveness of the AgNW-PEG composite material as an elastic conductor.

## 1. Introduction

Recently, flexible electronic devices capable of transducing physical phenomena, such as pressure, strain, and temperature, into electrical signals have received considerable attention for use in next-generation wearable electronics for health monitoring [1,2,3,4,5,6,7,8]. Particularly, a number of research groups have been pursuing the development of high-performance pressure sensors with high flexibility, optical transparency, and ultrahigh sensitivity, because of their wide range of potential applications in robotics and medicine, and applications to specific devices including smart phones, touch screen devices, and electronic skin [9,10,11,12,13,14,15,16,17]. Various types of pressure sensors exist, and are typically categorized by their transduction mechanisms. In this article, we focus on the piezoresistive transducing type (which has a number of advantages over the piezoelectric), capacitance, and triboelectric types (including low cost, convenient readout, faster response time, lower power consumption, small temperature dependence, and simple device structure) [18,19,20,21]. Most piezoresistive pressure sensors have simple sandwich structures in which conductive materials are embedded into insulated elastomeric polymer matrices. The elastic material is an essential component of such devices to accomplish high sensitivity and stable contact in the ultralow pressure region [22,23,24]. Therefore, it is reasonable to expect that properties of the elastic matrix play an important role in the performance of pressure sensors.

Several research groups have been utilized either polydimethylsiloxane (PDMS) or polyurethane (PU) as elastic matrices for highly sensitive, flexible pressure sensors, allowing tailoring of their shapes and structures [16,25,26,27,28,29]. However, elastic matrices, such as PDMS and PU, have the major limitation of poor processability (poor adhesion) originating from their low surface energies, making these polymers difficult to use in practical applications [30,31]. Contrastingly, elastic hydrogels having highly porous and three-dimensional polymer networks have significantly lower elastic modulus and good flexibility similar to that of natural tissues, making them more suitable for use as elastic matrices in pressure sensing applications [23,32,33,34,35]. Several studies have demonstrated the use of conductive composite hydrogels to improve sensitivity while maintaining good reproducibility in pressure/strain sensors [22,36,37].

Herein we describe the development of a novel type of transparent, flexible, and highly sensitive piezoresistive pressure sensor based on silver nanowires (AgNWs) as the conductive material and PEG hydrogel as the elastic matrix. We chose to use these materials because (1) AgNWs have excellent electrical properties, flexibility, and mechanical robustness, and their use allows cost-efficient solution processing [38,39,40,41,42,43]; and (2) PEG allows a simple patterning process including control over shape and dimension [44,45,46,47]. An important design criterion for us was to construct a simple but powerful active layer in which highly conductive materials were embedded into soft hydrogel matrices. Our experiments showed that this piezoresistive pressure sensor based on the silver nanowires and poly(ethylene glycol) (AgNW-PEG) sandwich structure exhibited good electrical properties, excellent flexibility, good transparency, high sensitivity, and fast response time.

## 2. Materials and Methods

### 2.1. Materials

Silver nanowires of diameter 20–40 nm and length 20–30 μm were purchased from NANOPYXIS (Jeonju-si, Korea). Poly(ethylene glycol) diacrylate (PEG-DA; MW 575) and polyethylene terephthalate (PET; film thickness 0.175 mm) were purchased from Aldrich Chemicals (Gillingham, UK) and used without further purification. 4-(2-Hydroxyethoxy) phenyl-(2-hydroxy-2-propyl) ketone (Irgacure 2959) was purchased from BASF (Ludwigshafen, Germany). 3-Acryloxy-propyl trichlorosilane was obtained from Gelest, Inc. (Morrisville, PA, USA). Toluene and ethanol were purchased from Duksan Pure Chemicals Company Co., Ltd. (Ansan, Korea). Phosphate-buffered saline solution (PBS) was purchased from Life Technologies (Shanghai, China).

### 2.2. Preparation of AgNW-PEG Patterns on a Flexible PET Substrate

AgNW solutions of various concentrations (1.0, 0.8, 0.6, 0.4, and 0.2 wt %; 800 μL) were spin-coated onto glass substrates (50 mm × 50 mm) at 500 rpm for 30 s. Into PBS containing 1% *w*/*v* of a photoinitiator (Irgacure 2959), dissolved at 70% *v*/*v* in ethanol, PEG-DA (MW 575) was mixed to achieve a 60% *w*/*v* gel precursor solution. The PEG precursor solution (10 μL) was dropped onto the AgNW-coated glass and then covered with silane-treated PET. Silane modification was used to anchor the gel layer to the PET. The AgNW-coated glass was covered with silane-treated PET and then exposed to an ultraviolet (UV) light source (INNO Cure 2000; 2.32 mW·cm^−2^) through a photomask for 2 s. The UV-exposed region of the AgNW film was transferred to the silane-treated PET substrate along with the PEG hydrogel. Finally, the AgNW-PEG patterned PET was dipped into ethanol to remove the unexposed PEG precursor solution.

### 2.3. Characterization of Morphology and Transmittance of AgNW-PEG Patterns

The morphologies of the AgNW-PEG patterns were characterized using optical microscopy (Nikon eclipse Ti-S, Nikon Inc., Tokyo, Japan) and field emission scanning electron microscopy (FE-SEM, Hitachi, model S-4200, Carl Zeiss, model Merlin, Hitachi, Ltd., Tokyo, Japan). To measure the transparency of AgNW-PEG patterns, prepared using various AgNW concentrations (1.0, 0.8, 0.6, 0.4, or 0.2 wt %), ultraviolet-visible-near infrared (UV-vis-NIR) transmittance spectra of samples in the hydrogel state, immersed in water, were collected using a CARY 300 Bio spectrophotometer (Varian, Inc., Milpitas, CA, USA).

### 2.4. Measurements of Electrical Properties and Bending Stability

To measure sheet resistance, AgNW-PEG patterns (width 1 mm) were prepared on PET substrates by using AgNW solutions of various concentrations (1.0, 0.8, 0.6, 0.4, and 0.2 wt %) and using the PEG photolithography procedure described above. The measurement was performed by using a sheet resistance tester (CMT-100S, Advanced Instrument Technology, Suwon, Korea). Sheet resistance values were calculated as the averages of measurements from several different positions. To test the stability of patterned samples transferred to a flexible substrate and their performance under bending, I–V curves of patterned samples were tested before and after substrate transfer and under bending. The patterned samples were of AgNW (0.6 wt %)-PEG, and had line patterns 1 mm in width and 16 mm in length, with 4 mm × 3 mm squares at both ends, and measurements were taken using a two-point probe method under voltage from −1 to +1 V, using a PGSTAT204 electrical measurement device (Metrohm Autolab, Utrecht, The Netherlands). In a bending stability test, a patterned sample of AgNW (0.6 wt %)-PEG was bent to the bending radius of 10 mm on both sides of the substrate and then unbent.

### 2.5. Fabrication of Resistive Pressure Sensor Device Using AgNW-PEG Electrodes on PET

Resistive pressure sensors of patterned AgNW-PEG were fabricated on flexible PET substrates using a sandwich structure. Specifically, to fabricate each pressure sensor device, two AgNW-PEG electrodes with the pattern width of 1 mm were assembled face to face, putting them in contact with each other. The working and counter electrodes were connected to these electrodes. I–V curves of these devices were obtained to demonstrate their behavior as pressure sensors under a range of external pressures. To measure the current change of each sandwich pressure sensor, a potential was applied from an electrochemical analysis device using a chronoamperometry technique (potential: 1 V). The relative change of resistance (RCR) of each device ((R − R_0_)/R_0_) was calculated by using the resistance value obtained from the corresponding I–V curve.

## 3. Results and Discussion

**Figure 1:**
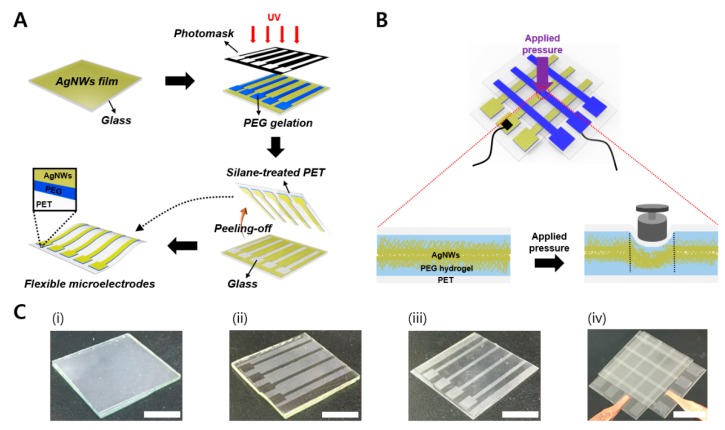
Schematic illustration of the fabrication of a resistive pressure sensor composed of elastic silver nanowires and poly(ethylene glycol) (AgNW-PEG) composite hydrogel patterned on flexible polyethylene terephthalate (PET). (**A**) Fabrication of flexible AgNW-PEG patterns on PET substrate via PEG photolithography. (**B**) Schematic illustration of the structural elasticity of the AgNW-PEG sandwich structure in the resistive pressure sensor. (**C**) Photographs of (i) AgNW film on glass, (ii) AgNWs left on glass after PEG photolithography, (iii) AgNW-PEG composite patterns on PET substrate, and (iv) resistive pressure sensor device based on AgNW-PEG sandwich structure (1 mm line pattern width; scale bars: 1 cm).

The process used to fabricate piezoresistive pressure sensors comprising patterned AgNW-PEG on flexible PET is schematically illustrated in Figure 1, including step-by-step photographs. A slightly modified version of a PEG photolithography process described in our previous works [48,49,50] was employed to construct each active layer of AgNW-PEG. In contrast to our previous studies the objective of the present work was to directly study the influence of AgNWs and PEG hydrogel upon the performance of contact resistive pressure sensors.

As shown in Figure 1A, UV-induced PEG gelation at the AgNW-PEG interface through a photomask triggered a strong crosslinking reaction between the AgNW network structures and PEG polymer chains. As a result, AgNWs were cleanly transferred from the glass to the PET substrate in the UV exposed regions, along with the PEG hydrogel (Figure 1C(iii)). As shown in Figure 1B, we fabricated resistive pressure sensors based on the AgNW-PEG sandwich structure and then investigated the effects of using the elastic AgNW-PEG composite hydrogel upon the performance of the pressure sensor, as discussed below.

**Figure 2:**
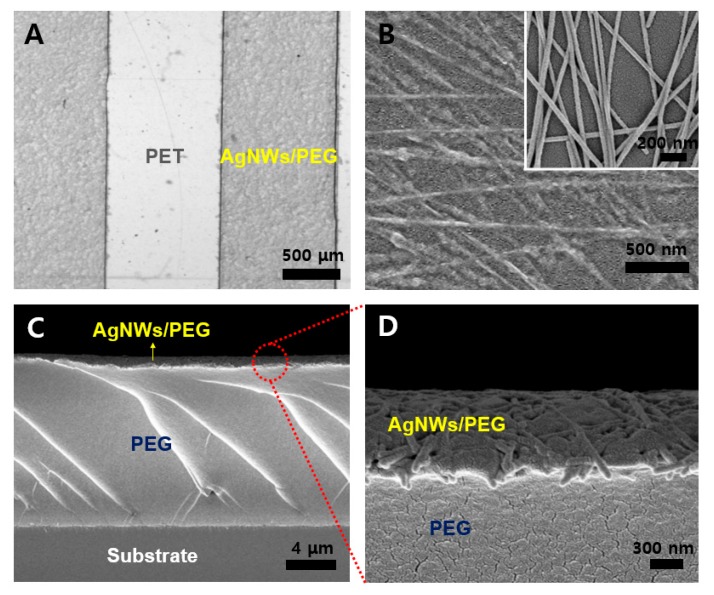
(**A**) Optical micrographs of AgNW-PEG patterns of line width 1 mm on PET. (**B**) FE-SEM micrographs of AgNW-PEG patterns on a PET substrate. (B inset) SEM micrograph of AgNW patterns on glass. (**C**,**D**) Cross sectional views of AgNW-PEG patterns of line width 1 mm on substrate.

Optical and scanning electron microscope (SEM) micrographs showed that the AgNW-PEG patterns were successfully fabricated on the PET substrate via PEG photolithography (Figure 2). Tiled cross-sectional SEM micrographs clearly showed that each active layer of AgNW-PEG was composed of two distinct regions (AgNWs embedded in PEG, and pure PEG), which work together as an elastic conductor (Figure 2C,D). In contrast to pure AgNW patterns without PEG hydrogel on glass (Figure 2B inset), highly magnified SEM micrographs of a region of AgNWs embedded in PEG showed that AgNW networks were embedded in the PEG surface layer, leading to good stability and flexibility of the elastic AgNW-PEG composite hydrogel (Figure 2B,D). This structure of AgNWs embedded in PEG likely arose from the ability of the PEG precursor solution to easily penetrate into AgNW networks during UV-induced PEG gelation.

**Figure 3:**
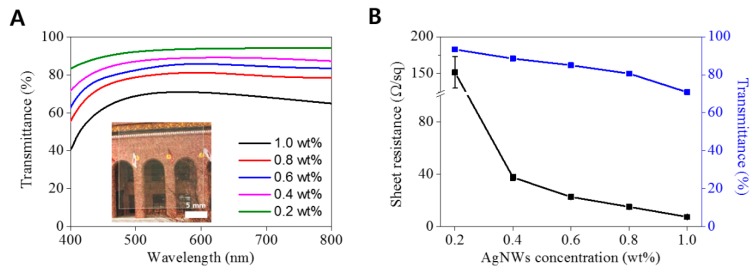
(**A**) Optical transmittance of AgNW-PEG layer on PET films prepared using various concentrations of dispersed AgNWs in the precursor solutions. (A inset) Photograph of highly transparent AgNW-PEG line patterns of line width 1 mm on PET film (AgNW concentration: 0.6 wt %). (**B**) Sheet resistance and transmittance at 550 nm of AgNW-PEG on PET film, versus AgNW concentration of the precursor used.

We investigated the transmittance and the sheet resistance of elastic AgNW-PEG composite hydrogels prepared using various AgNW concentrations; the transmittance and sheet resistance of AgNW-PEG composites decreased with increasing AgNW concentration from 0.2% to 1.0% (Figure 3A,B). AgNW-PEG patterns of 0.6 wt % AgNWs showed high transmittance, with T_550nm_ of around 86% (Figure 3A inset), and good electrical properties with the low sheet resistance of 22.69 Ω·sq^−1^ (conductivity: 15 × 10^3^ S·cm^−1^). Therefore, the 0.6 wt % AgNW formulation of elastic AgNW-PEG composite hydrogel was chosen for further experiments related to bending durability, sensitivity, and response speed of transparent resistive pressure sensors.

**Figure 4:**
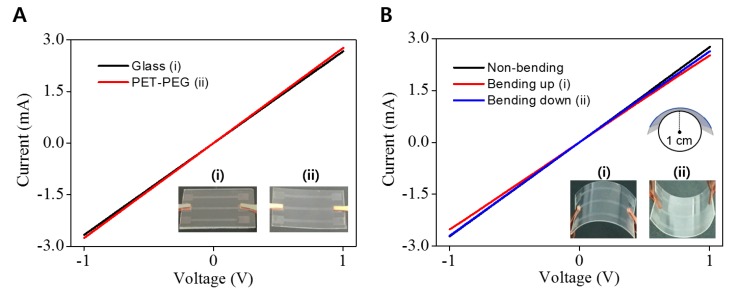
(**A**) Current–voltage (I–V) characteristics of AgNW-PEG line patterns (i) on glass before the direct transfer process, and (ii) on PET substrate after direct transfer (0.6 wt % AgNW concentration). (**B**) I–V curves of AgNW-PEG line patterns on PET during a bending test (0.6 wt % AgNW concentration; 1 cm bending radius).

We next evaluated the I–V characteristics of AgNW-PEG patterns on glass and PET substrates, respectively before and after PEG photolithography. Nearly identical I–V characteristics were observed on the glass and PET substrates (Figure 4A), indicating that the PEG photolithography process allowed intact AgNW transfer from glass to flexible PET film. To further evaluate the mechanical flexibility of AgNW-PEG composite hydrogel, we examined changes in I–V characteristics under folding and bending. AgNW-PEG showed very similar I–V curves in the flat state and under bidirectional bending at the bending radius of 1 cm (Figure 4B), suggesting its good bending durability as an electrode.

**Figure 5:**
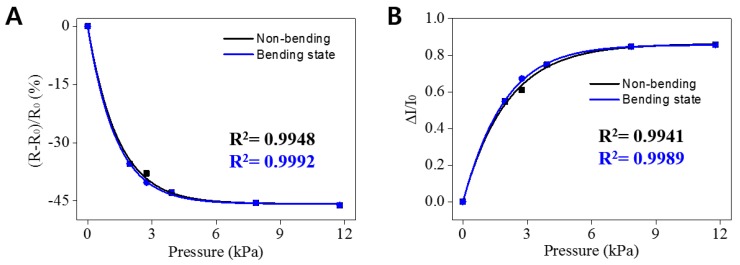
Responses of a sandwich-type pressure-responsive sensor device of elastic AgNW-PEG composite hydrogel patterns on flexible PET (1 mm line pattern width). (**A**) Relative resistance change (RCR), and (**B**) relative current change (RCC) of the sensor with bending or non-bending state under various pressures (1.96, 2.75, 3.92, 7.85, and 11.77 kPa, 1.5 cm bending radius).

We constructed a transparent and flexible resistive pressure sensor device composed of a sandwich structure of the elastic AgNW-PEG composite (Figure 1B,C). Relative change of current (RCC) and the resistance (RCR) values ((R − R_0_)/R_0_) were calculated using resistances obtained from the I–V data collected in bending or non-bending state under various pressures. There was no distinct difference between the RCR and RCC values in both states, indicating that the sensor can detect pressure even in the bending state. These data were in line with I–V curve results shown in Figure 4B. The RCR values with bending or non-bending state changed greatly under increasing pressures from 0 to 11.77 kPa (1.96, 2.75, 3.92, 7.85, and 11.77 kPa; Figure 5A,B). It needs to be noted that there was no significant difference in the values of current and resistance at pressures higher than 12 kPa.

The elastic AgNW-PEG based pressure sensor exhibited higher sensitivity (S = *d*R/*d*P; R, resistance; P, applied pressure) of ~69.7 and ~1.9 kPa^−1^ in the respective pressure ranges of 0–1.96 and 1.96–7.85 kPa, which were comparable to previous reports [36,37,51,52,53]. The pressure detection threshold of this sensor was determined to be around ~2 kPa.

**Figure 6:**
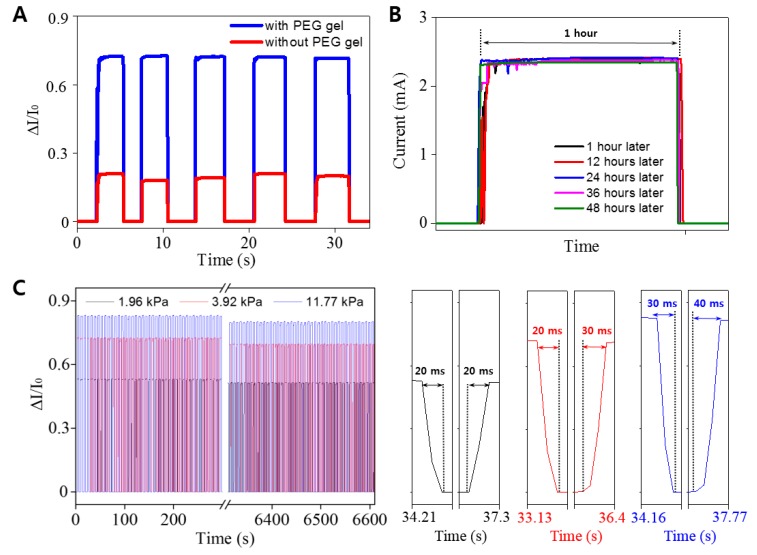
(**A**) Pressure sensing tests (pressure: 3.92 kPa) of resistive pressure sensors based on AgNW-PEG composite (blue line) and pure AgNW without PEG (red line). (**B**) Chronoamperometry curve of AgNW-PEG based pressure sensor under 3.92 kPa for 48 h. To demonstrate drift stability, measurements were conducted for 1 h at 1, 12, 24, 36, and 48 h after exercise. (**C**) Cyclability and response time curves of resistive pressure sensor based on AgNW-PEG composite hydrogel under several pressures (1.96, 3.92, and 11.77 kPa).

It is important to note that the AgNW-PEG composite based pressure sensor presented herein had much better sensitivity than a sensor based on a layer of pure AgNWs on a rigid substrate, demonstrating the effectiveness of the elastic PEG hydrogel to enhance pressure sensitivity (Figure 6A).

The electromechanical sensors typically exhibit drift phenomena that are less accurate and less sensitive when exposed to steady stimuli. Therefore, we investigated the current difference of AgNW-PEG based pressure sensor under 3.92 kPa for 48 h as shown in Figure 6B. As a result, the initial current value was decreased to only 1.8%, indicating the good stability of AgNW-PEG based pressure sensor. Other important requirements for high-performance pressure sensors are excellent cyclability and fast response time. Our pressure sensor produced stable and reproducible signals with very fast response and relaxation times (20–40 ms) over 1000 cycles of pressure loading/unloading (1.96, 3.92, and 11.77 kPa) (Figure 6C). These results likely arose from the stable electrical contact and excellent elastic deformation of the AgNW-PEG composite hydrogel under static loading.

**Figure 7:**
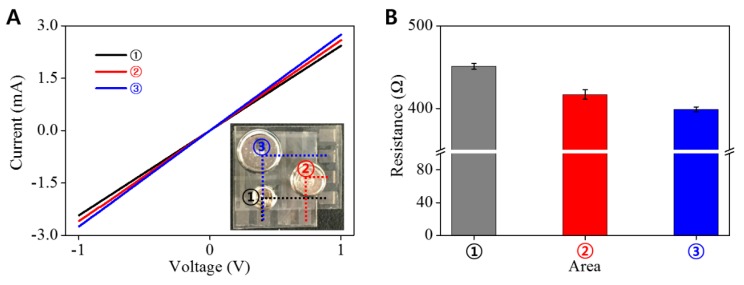
(**A**) The I–V curve and (**B**) resistance of a sandwich-type pressure-sensor device for demonstrating the functionality of the sensor array. (A inset) Photograph of sensor array with AgNW-PEG line patterns (width: 1 mm) on PET substrate under several weight pressures (① 2 g; ② 5 g; ③ 10 g).

To investigate the spatial distinguishable ability of pressure sensor array, three weights (2, 5, 10 g) were placed on the marked sites of the 4 × 4 pixels sensing array (each is 1 mm × 1 mm, Figure 7A inset). Figure 7 clearly shows the change in I–V curves depending on weights as well as the resistance decrease by increasing weight. These results indicate that this sensor array based on AgNW-PEG composite patterns can be used to detect the distribution of the applied pressure with a high sensitivity. Overall, it is clear that the AgNW-PEG composite patterns on flexible PET substrate described herein can enhance our ability to develop highly transparent, flexible, and ultrasensitive pressure sensors.

## 4. Conclusions

We have reported a resistive pressure sensor with high performance, including good transparency, high flexibility, excellent sensitivity, and fast and stable response. A key design consideration for this pressure sensor was to construct an active electrode layer composed of highly conductive AgNWs and elastic PEG hydrogel. A simple PEG photolithography can be employed to pattern elastic AgNW-PEG composite hydrogels on flexible PET film. A resistive pressure sensor based on a sandwich structure of the AgNW-PEG composite had high transmittance (including 86% transmittance at 550 nm), good flexibility (only 5.8% resistance increase under bending to the radius of 10 mm), high sensitivity (up to 69.7 kPa^−1^), low sensing threshold (~2 kPa), and fast response time (20–40 ms). We foresee that the proposed strategy to fabricate elastic composite conductors could enable diverse technologies for the development of next-generation bioelectronics systems.
